# An Interdisciplinary Study of Lysozyme Interactions with Hexacyanoferrate(III)/(II) Ions

**DOI:** 10.3390/ijms26178511

**Published:** 2025-09-02

**Authors:** Ola Grabowska, Krzysztof Żamojć, Anna Kloska, Paweł Niedziałkowski, Sergey A. Samsonov, Dariusz Wyrzykowski

**Affiliations:** 1 Faculty of Chemistry, University of Gdańsk, Wita Stwosza 63, 80-308 Gdańsk, Poland; krzysztof.zamojc@ug.edu.pl (K.Ż.); pawel.niedzialkowski@ug.edu.pl (P.N.); sergey.samsonov@ug.edu.pl (S.A.S.); 2 Faculty of Biology, University of Gdańsk, Wita Stwosza 59, 80-308 Gdańsk, Poland; anna.kloska@ug.edu.pl

**Keywords:** lysozyme, hexacyanoferrate(III)/(II) ions, binding interactions, enzymatic activity

## Abstract

In this article, the binding interactions of lysozyme with hexacyanoferrate(III)/(II), i.e., [Fe(CN)_6_]^3−^ and [Fe(CN)_6_]^4−^ ions, have been characterised using steady-state fluorescence spectroscopy (SF), isothermal titration calorimetry (ITC), circular dichroism spectroscopy (CD), cyclic voltammetry (CV), and molecular-dynamics-based computational approaches. Studies have shown that under experimental conditions (10 mM cacodylate buffer, pH 7, 298.15 K), complexes with a 1:1 stoichiometry are formed. Four distinct regions on the lysozyme surface patches with the potential to bind hexacyanoferrate(III)/(II) were identified and described. Thermodynamic parameters revealed that the interactions are predominantly governed by electrostatic and van der Waals forces. These interactions enhance the electron transfer kinetics of the [Fe(CN)_6_]^3−/4−^ system. The secondary structure of the protein is not affected by these interactions. Enzyme activity studies demonstrated that the affinity of lysozyme for the substrate remained unchanged regardless of whether free lysozyme or the lysozyme-[Fe(CN)_6_]^3−/4−^ complex was present in the test sample. Finally, biological tests performed on both Gram-positive (*B. subtilis*, *S. aureus*) and Gram-negative (*E. coli*, *P. aeruginosa*) bacteria confirmed the results of the biochemical analysis, indicating that [Fe(CN)_6_]^3−/4−^ ions do not block the active site of the enzyme and do not interfere with its activity.

## 1. Introduction

Hexacyanoferrate(III) ([Fe(CN)_6_]^3−^) and hexacyanoferrate(II) ([Fe(CN)_6_]^4−^) ions are among the most thermodynamically stable cyanide complexes. They are characterised by their symmetrical octahedral geometry, the high negative charge of the complex anion and the ability to reversibly change the oxidation state of the coordination centre. From a kinetic point of view, these complexes can be classified as inert, exhibiting a slow exchange of CN^−^ ligands under certain conditions, namely high temperature and acidity. The specific physicochemical properties of hexacyanoferrate(III)/(II) ions make these coordination units useful in a wide range of chemical applications, including electrochemistry, the pigment industry and analytical and diagnostic chemistry [1,2,3,4]. As protons are not involved in the oxidation/reduction equation, the reduction potential of the [Fe(CN)_6_]^3−^/[Fe(CN)_6_]^4−^ redox couple (E^0^ *ca*. 0.41 V) remains relatively stable across a broad pH range, specifically from 4 to 13. The [Fe(CN)_6_]^3−^ ion is a relatively weak oxidant with selective activity towards the most readily oxidisable substrates [5,6]. For this reason, it is often used as a scavenger and has the potential to remove contaminants from the system [7]. Hexacyanoferrate(III) ions are also present in some ecosystems as a result of anthropogenic human activities. Particularly high concentrations of these cyanocomplexes are found in wastewater from gold mining, which is associated with the use of cyanide ions in the leaching of gold from minerals [8]. The high affinity of hexacyanoferrate(III)/(II) ions for certain metals prevents their distribution in the natural environment [9,10]. It is interesting to note that hexacyanoferrate(II) in the form of Prussian blue, Fe_4_[Fe(CN)_6_]_3_, has also been used as an oral agent to increase the excretion of caesium-137 and thallium from the body. It has been shown that the accumulation of Fe_4_[Fe(CN)_6_]_3_ in the intestine and its interaction with caesium ions disrupt the intestinal-hepatic circulation of radioactive ions, thereby reducing the radioactive burden on the body [11]. However, it should be noted that the presence of exogenous oxidising compounds, such as hexacyanoferrate(III)/(II) ions, has important implications for the course of redox processes. The [Fe(CN)_6_]^3−^ ion has been shown to act as a mild selective oxidant by reacting with glutathione, affecting its redox state, and thereby interfering with its function by impairing its activity in cellular defence against oxidative stress [12].

To the best of our knowledge, little attention has been given to the interactions between proteins and these common cyanocomplexes, which can serve as an excellent model for small molecules with a high negative charge. In recent studies, we have demonstrated that the affinity of hexacyanoferrate(III)/(II) ions for two globular proteins, namely human serum albumin (HSA) and bovine serum albumin (BSA), plays a key role in the bioaccumulation and transport of these species in biological systems. The formation of relatively stable albumin-hexacyanoferrate(III)/(II) complexes at the Sudlow II site effectively blocks this binding site [13]. Consequently, this inhibits the interaction of weaker small-molecule ligands in this region of the protein. To gain a deeper insight into the interactions between proteins and hexacyanoferrate(III)/(II) ions, we have extended our research in this area by focusing on the analysis of interactions with hen egg white lysozyme (HEWL), which contains six tryptophan residues (each can be differently accessible to quenchers—exposed or buried—because they are placed in distinct environments), several of which are known to be near the action site [14]. Understanding the physicochemical principles of these interactions is crucial to understanding the factors that influence the biological activity of lysozyme, which has been identified as a natural product with antiviral [15] and antibacterial [16] properties and is widely used in the life sciences [15]. In addition, HEWL is particularly interesting for the study of protein–ion interactions, especially with negatively charged ions. In contrast to previously studied proteins (HSA, BSA), it has a positive charge (at pH 7), a smaller size and a relatively rigid structure compared to albumins.

## 2. Results and Discussion

### 2.1. Binding Analysis of Hexacyanoferrate(III)/(II) Ions to Lysozyme

**Figure 1:**
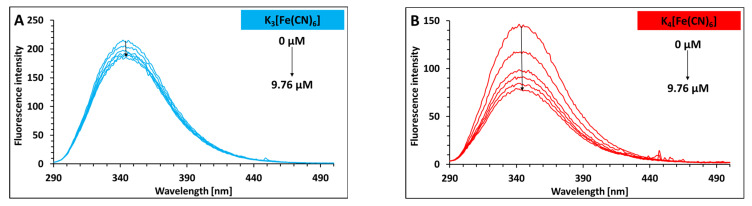
The fluorescence emission spectra of free lysozyme and its mixtures with K_3_[Fe(CN)_6_] (**A**); The fluorescence emission spectra of free lysozyme and its mixtures with K_4_[Fe(CN)_6_] (**B**). Concentration range: 0–9.76 µM; in 10 mM Caco buffer of pH 7; λ_ex_ = 275 nm; c _LYS_ = 1.76 µM; 298.15 K.

The intrinsic fluorescence changes in lysozyme in the presence of increasing concentrations of cyanocomplexes have been studied to understand the effect of the hexacyanoferrates (III)/(II) on the tryptophan environment. After being excited at 275 nm, lysozyme dissolved in 10 mM Caco buffer at pH 7 shows a single emission peak with a maximum at 345 nm. When K_3_[Fe(CN)_6_] and K_4_[Fe(CN)_6_] are added, the fluorescence intensity decreases with no significant shift in the observed band (Figure 1). These findings imply that K_3_[Fe(CN)_6_] and K_4_[Fe(CN)_6_] can bind to the protein, with a negligible change in the tryptophan’s environment. This indicates that those tryptophan residues emitting at larger wavelengths are not quenched more readily than the shorter-wavelength tryptophans [14].

**Figure 2:**
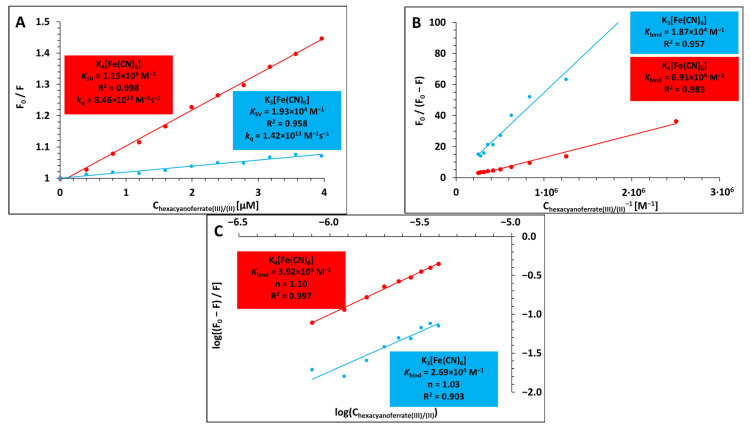
Stern–Volmer plots for the steady-state fluorescence quenching of lysozyme by K_3_[Fe(CN)_6_] and K_4_[Fe(CN)_6_] (**A**); The plots of $\frac{F_{0}}{F_{0}-F}$ as a function of [Q]^−1^ for the steady-state fluorescence quenching of lysozyme by K_3_[Fe(CN)_6_] and K_4_[Fe(CN)_6_] (**B**); The plots of $log\left[\frac{F_{0}-F}{F}\right]$ as a function of $log\left[Q\right]$ for the steady-state fluorescence quenching of lysozyme by K_3_[Fe(CN)_6_] and K_4_[Fe(CN)_6_] (**C**). All experiments were performed in 10 mM Caco buffer of pH 7; λ_ex_ = 275 nm; λ_em_ = 345 nm; c _LYS_ = 1.76 µM; 298.15 K.

The Stern–Volmer equation was initially used to analyse the fluorescence quenching data in order to gain a better understanding of the qualitative and quantitative modes of the interactions between lysozyme and the hexacyanoferrates(III)/(II):

(1) $$\frac{F_{0}}{F}=1+K_{SV}\left[Q\right]=1+k_{q}\tau_{0}\left[Q\right],$$

where [Q] is the quencher concentration, *K*_SV_ is the Stern–Volmer quenching constant, *k*_q_ is the bimolecular quenching rate constant, *τ*_0_ is the average lifetime of the lysozyme in the absence of a quencher, and F_0_ and F represent the steady-state fluorescence intensities in the absence and presence of a quencher (K_3_[Fe(CN)_6_] and K_4_[Fe(CN)_6_]), respectively [14]. Plots of $\frac{F_{0}}{F}$ = f([Q]) should be linearly dependent on quencher concentration in the case of either purely static or purely dynamic quenching [17]. The Stern–Volmer plots in our experiments for the quenching of lysozyme by K_3_[Fe(CN)_6_] and K_4_[Fe(CN)_6_] showed a downward curvature (towards the x-axis) and were non-linear in the initially selected concentration range of the quenchers (0–9.76 µM), which is a characteristic feature of two fluorophore populations, one of which is not accessible to the quencher [14]. This could mean that the quencher can only reach the fluorescent residues on the outer surface (polar or charged quenchers cannot penetrate the hydrophobic interior of the protein) [18]. Therefore, all of the fluorescence quenching experiment results are shown in the next section of the manuscript for the concentration range of K_3_[Fe(CN)_6_] and K_4_[Fe(CN)_6_] from 0 to 3.96 µM, which corresponds to a final hexacyanoferrate/protein molar ratio of 2.25. The fluorescence quenching of tryptophan residue in lysozyme by K_3_[Fe(CN)_6_] and K_4_[Fe(CN)_6_] is shown in Figure 2A (as Stern–Volmer plots) along with Stern–Volmer quenching constants (*K*_SV_), linear correlation coefficients (R^2^), and the newly found values of the bimolecular quenching rate constants (*k*_q_). The latter were computed using the average fluorescence lifetime of free lysozyme, which is roughly 1.36 ns (at pH 8.0 phosphate buffer) [19]. Strictly straight lines with no appreciable departures from the observed linearity were obtained in the investigated narrow concentration range. The newly calculated bimolecular quenching rate constant values were contrasted with the maximum value for collisional quenching, which is 2.0 × 10^10^ M^−1^ s^−1^ for interactions of different quenchers with biopolymers in aqueous media [20]. The estimated values of *k*_q_ for each system studied are much higher than the limiting diffusion rate constant (by about three orders of magnitude). Ground-state complex formation was confirmed to play a major role in the systems under investigation, whereas dynamic collisions were found to play a negligible role, as bimolecular quenching rate constant values are thought to be decisive in distinguishing between dynamic and static quenching mechanisms [21,22,23,24].

Given that the fluorescence quenching of lysozyme by K_3_[Fe(CN)_6_] and K_4_[Fe(CN)_6_] is a static quenching process, the following equations [25,26] were used to create appropriate plots (Figure 2B,C) and calculate the binding constants (*K*) of the newly formed complexes and the number of binding sites:

(2) $$\frac{1}{F_{0}-F}=\frac{1}{F_{0}}+\frac{1}{{K\cdot F}_{0}\left[Q\right]},$$

(3) $$log\left[\frac{F_{0}-F}{F}\right]=logK+n\cdot log\left[Q\right].$$

The binding constant values as well as the number of binding sites for both of the protein/hexacyanoferrate(III)/(II) systems under investigation were derived from the regression equations used for these curves and are gathered in Table 1.

**Figure 3:**
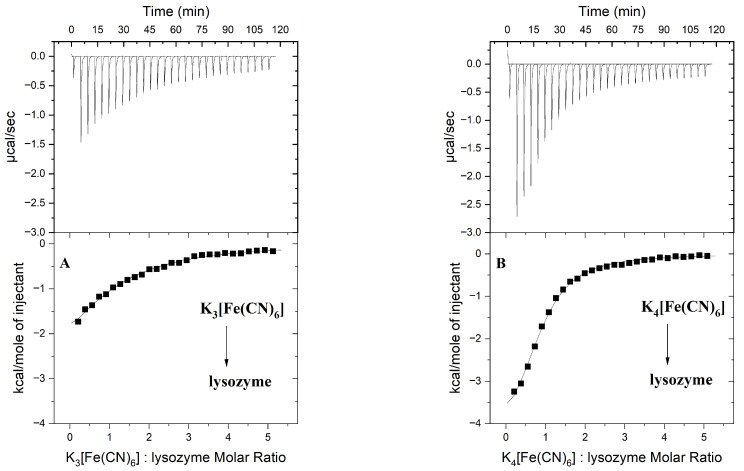
Calorimetric titration isotherm of the binding interactions of K_3_[Fe(CN)_6_] with lysozyme (**A**); calorimetric titration isotherms of the binding interactions of K_4_[Fe(CN)_6_] with lysozyme (**B**). Experiments were performed in the 10 mM Caco buffer of pH 7, at 298.15 K.

Along with steady-state fluorescence spectroscopy, isothermal titration calorimetry (ITC) was used to provide a complete thermodynamic characterisation of the interactions studied. The parameters of the interactions, including the stoichiometry of the resulting protein–ligand complexes, binding constants, and thermodynamic parameters, are summarised in Table 1, while the representative binding isotherms for the lysozyme–hexacyanoferrate(III)/(II) system are shown in Figure 3.

The ITC data were processed by fitting binding isotherms to the equilibrium model that assumes a single set of identical binding sites. The validity of the fitting model used was previously confirmed by the results of the SF, which revealed the presence of one site on the lysozyme surface capable of binding [Fe(CN)_6_]^3−^ and [Fe(CN)_6_]^4−^ complexes (Table 1).

**Figure 4:**
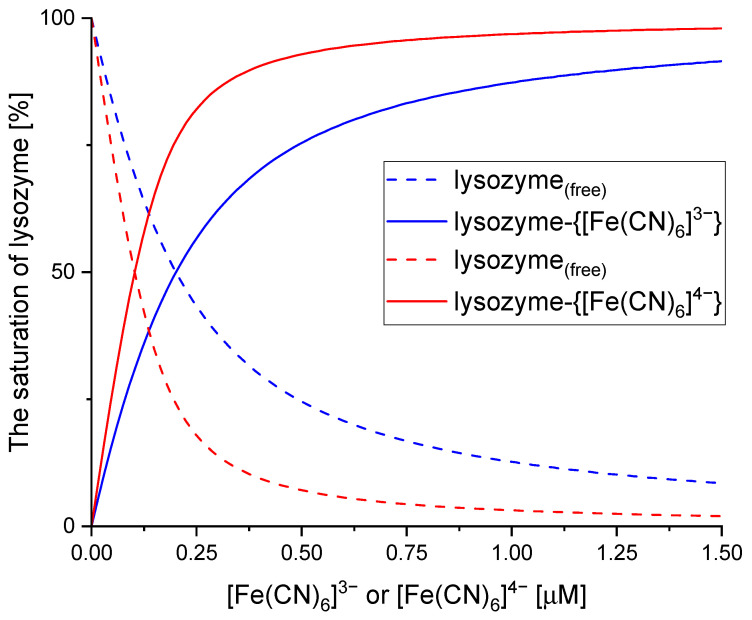
The relationship between the saturation fraction of lysozyme (θ) and the concentration of free ligands ([Fe(CN)_6_]^3−^ or [Fe(CN)_6_]^4−^).

The species distribution diagrams that represent the percentage of a free protein ([lysozyme]) and the fraction of a protein bound to a ligand ([lysozyme]-ligand) are shown in Figure 4. The saturation fraction, $\theta =\alpha_{lysozyme-ligand}=\frac{\left[lysozyme-ligand\right]}{c_{lysozyme}}$ (where α_lysozyme-ligand_ denotes the fraction of a protein bound to a ligand; c_lysozyme_ is the total concentration of the protein), quantifies the extent to which the binding site is occupied by [Fe(CN)_6_]^3−^ and [Fe(CN)_6_]^4−^ and reflects the dynamic equilibrium between the free and bound states of the protein–ligand complex. According to the SF and ITC results (Table 1), it has been proven that [Fe(CN)_6_]^4−^, which carries a higher negative charge, exhibits stronger interactions with lysozyme compared to [Fe(CN)_6_]^3−^. Consequently, this leads to more efficient protein saturation when bound to [Fe(CN)_6_]^4−^, highlighting the crucial influence of charge in regulating binding affinity and saturation efficiency (Figure 4).

Thermodynamic parameters of the resulting protein–ligand complexes indicate that the interactions are mainly governed by the enthalpy factors |ΔH| > |TΔS|. The negative binding enthalpies confirm the pivotal role of electrostatic and van der Waals forces in the binding processes and simultaneously explain a slightly higher affinity of the positively charged protein for [Fe(CN)_6_]^4−^ than for [Fe(CN)_6_]^3−^. The observed slight increase in entropy values can be attributed to the dehydration of the reactants and/or ion exchange occurring at the binding site of the hexacyanoferrate(III)/(II) ions with the protein. However, circular dichroism studies showed that the observed affinity of [Fe(CN)_6_]^3−^ and [Fe(CN)_6_]^4−^ for lysozyme, although mainly governed by electrostatic interactions, had little effect on changes in the secondary structure of lysozyme, despite the different charges of the cyanocomplexes (Figure S1 and Table S1).

It is important to note that the total charge of a protein does not serve as a straightforward determinant of its affinity for negatively charged ligands. In addition to the charge of the ligand, factors such as the spatial arrangement of the ligand, the presence of hydrophobic regions within the ligand structure, and the ligand’s size relative to the protein surface are also significant considerations. The observed differences in the hexacyanoferrate complexes are reflected in their binding affinities to lysozyme and albumin, namely human serum albumin (HSA) and bovine serum albumin (BSA). Previous studies have shown that both albumins have a single binding site for the [Fe(CN)_6_]^3−/4−^ complexes, with a stoichiometric binding ratio of 1:1 [13]. Significantly, the binding strength of these complexes to albumins is higher even though albumin has a net negative charge under the same experimental conditions (pH 7). In contrast, the interactions between [Fe(CN)_6_]^3−/4−^ and lysozyme revealed that the charge of the complexes affects the strength of the binding interactions with lysozyme. The situation is different for the interaction of [Fe(CN)_6_]^3−^ and [Fe(CN)_6_]^4−^ ions with BSA. Studies have shown a similar affinity of both ions for albumin, suggesting that the difference in charge between these ions does not affect their binding behaviour [13]. The possible explanation for this phenomenon is that albumin, as the primary transport protein in the bloodstream, has multiple binding sites that can accommodate a wide range of endogenous and exogenous compounds based on their shape, size or hydrophobicity. In addition, the structural flexibility of albumin facilitates conformational changes that enhance its ligand binding capacity. In contrast, the binding interactions of lysozyme tend to be more specific and less flexible. Since strong binding reduces the plasmatic distribution of free drug, while weak binding can result in a short lifetime or poor distribution, the value of the binding constant is especially important for understanding the drug’s distribution in plasma [27,28]. When compared to other well-known strong biomolecule–ligand complexes with binding constants ranging from 10^5^ to 10^8^ M^−1^ [29,30,31,32], the binding constant values obtained for the investigated lysozyme complexes indicate a moderate binding affinity to the protein for both hexacyanoferrates.

### 2.2. The Electrochemical Behaviour of Hexacyanoferrate(III)/(II) and Its Lysozyme Complexes

The [Fe(CN)_6_]^3−/4−^ ions are well known for their ability to reversibly accept or donate a single electron. It has been reported that the reduction potential of this redox couple depends on various environmental factors, including the type of solvent and the presence of metal ions in the system [33]. In this study, we have focused on the analysis of the effect of the experimental conditions (the type of buffer used) and the binding affinity of hexacyanoferrate(III)/(II) to lysozyme on the electrochemical behaviour of [Fe(CN)_6_]^3−/4−^.

The cyclic voltammograms of the investigated samples (vs. Ag/AgCl reference electrode) are presented in Figure S2. The separation between the anodic (E*_pa_*) and cathodic (E*_pc_*) peak potentials (ΔE*_p_* = E*_pa_* − E*_pc_*) was studied in two solutions, namely 0.1 M KCl and 0.1 M Caco buffer (pH 7). The first system was used as a reference solution because 0.1 M KCl is the most commonly used electrolyte for electrochemical analysis. The results showed a strong influence of Caco buffer on the cyclic voltammetry response. In the case of 0.1 M KCl, the ΔE*_p_* value was 82 mV, whereas in 0.1 M Caco buffer, the ΔE*_p_* value increased to 168 mV, indicating a decrease in kinetic barriers resulting in less favourable electron transfer in the Caco buffer. At the same time, it is noteworthy that the components of the Caco buffer are not redox-active in the given experimental potential range (Figure S2).

Interestingly, quite different behaviour was observed in the case of [Fe(CN)_6_]^3−/4−^ bound with lysozyme. The peak-to-peak separation (ΔE*_p_*) for the system containing [Fe(CN)_6_]^3−^ saturated with protein was found to be 67 mV after correction for dilution. In particular, the observed oxidation and reduction current peaks were comparable to those observed in the voltammograms of [Fe(CN)_6_]^3−/4−^ in 0.1 M KCl. The observed electrochemical behaviour indicates that the interaction between [Fe(CN)_6_]^3−/4−^ and the protein significantly enhances the electron transfer kinetics. This enhancement is evident when compared to the situation where hexacyanoferrate(III)/(II) ions are present in solution in an unbound (free) form.

### 2.3. Binding Sites of Hexacyanoferrate(III)/(II) Ions to Lysozyme

There are several binding sites on the lysozyme surface capable of binding small negatively charged species. Some of these sites are specific for lysozyme, while others are specific for the anion [34]. Lysozyme has been reported to bind polyanions such as DNA and RNA, both specifically and non-specifically [35]. Similar binding interactions were observed for small polyvalent anions, namely adenosine tripolyphosphate (ATP) and tripolyphosphate (TPP) [36].

**Figure 5:**
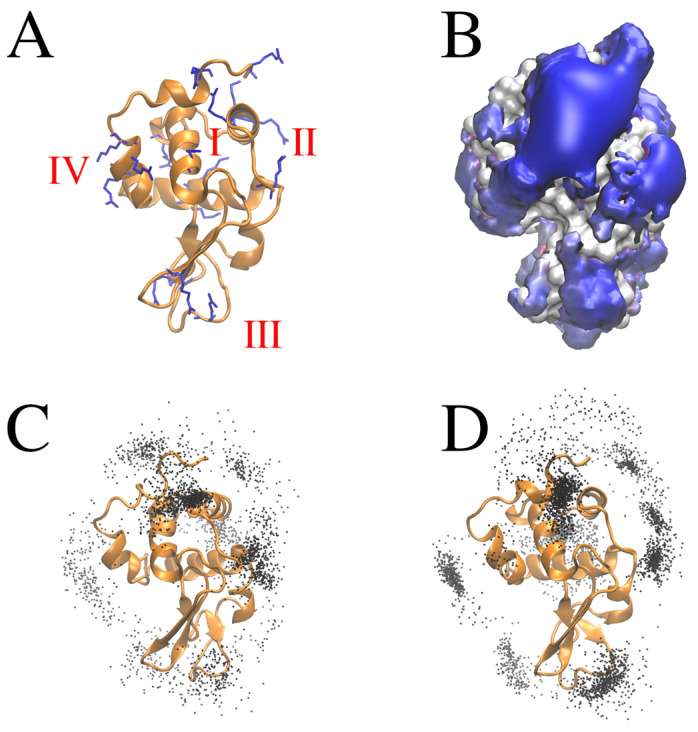
MD analysis of lysozyme interactions with the [Fe(CN)_6_]^3−^ and [Fe(CN)_6_]^4−^ anions. Experimental structure of chicken lysozyme (PDB ID: 193L). Positively charged residues are shown in blue sticks. Roman numbers (in red) correspond to the regions predicted to be involved in binding the [Fe(CN)_6_]^3−^ and [Fe(CN)_6_]^4−^ anions (**A**). An electrostatic potential isosurface of 2.5 kcal mol^−1^ e^−1^ calculated using the PBSA approach (**B**). The density of the centres of mass of ligands within 10.0 Å of protein visualised at a step of 1 ns: [Fe(CN)_6_]^3−^ (**C**); [Fe(CN)_6_]^4−^ (**D**).

To gain insights into the atomistic details that explain the differences in the binding of the [Fe(CN)_6_]^3−^ and [Fe(CN)_6_]^4−^ anions to the lysozyme, we employed molecular dynamics (MD) simulations. The MD simulations allowed the identification of four distinct regions that have the potential to bind the complexes (Figure 5). These regions are characterised by the presence of positively charged residues: Arg5, Lys33, Arg125 (region I); Lys1, Arg14 (region II); Arg45, Arg68 (region III); and Arg112, Arg114, Lys116 (region IV) (Figure 5A).

The observation that these regions are localised similarly, regardless of the charge of [Fe(CN)_6_]^3−^ or [Fe(CN)_6_]^4−^ on the surface patches corresponding to the highest positive electrostatic potential (Figure 5B), indicates that the interactions between the anions and lysozyme are predominantly governed by electrostatic forces. Furthermore, the more pronounced density of the points in space corresponding to the position of the ligand centres of mass during the MD simulation for [Fe(CN)_6_]^4−^ supports the crucial role of electrostatic interactions in this system (Figure 5C,D). These findings are consistent with the results of the calorimetric study. In the MD simulation, [Fe(CN)_6_]^4−^ forms more contacts with lysozyme compared to [Fe(CN)_6_]^3−^. These contacts are defined as those occurring within a cutoff distance of 10 Å between the centre of mass of a ligand molecule and any protein residue (Figure S3). Linear interaction energy (LIE) analysis also suggests that the interactions between the [Fe(CN)_6_]^4−^ and lysozyme are stronger than in the case of [Fe(CN)_6_]^3−^: there are ~1.5 more frames where the LIE energies for a protein–ligand pair were lower than −1 and −2 kcal mol^−1^. At the same time, the per residue contacts are very similar: the correlation between the time fractions of the contacts established by a residue for two different ligands is 0.79.

### 2.4. Lysozyme Activity

#### 2.4.1. Biochemical Studies

The active site of lysozyme is located in a cleft on the enzyme’s exterior. It is responsible for catalysing the hydrolysis of beta-(1,4)-glycosidic bonds between *N*-acetylglucosamine (NAG) and *N*-acetylmuramic acid (NAM) in the peptidoglycan layer of the bacterial cell walls. Two critical amino acid residues within this active site, glutamic acid 35 (Glu35) and aspartate 52 (Asp52), are essential for catalytic activity [37]. To assess how the binding interactions between lysozyme and hexacyanoferrate(III)/(II) ions affect the enzymatic activity of lysozyme, a synthetic fluorogenic substrate, 4-methylumbelliferyl *β*-D-*N*,*N*′,*N*″-triacetylchitotrioside (4-MUF-triNAG), was used. Lysozyme catalyses the hydrolysis of glycosidic bonds, resulting in the release of the fluorescent product, 4-methylumbelliferone (4-MUF). This is manifested by an increase in the fluorescence intensity, which directly correlates with the enzymatic activity of lysozyme. The enzymatic activities of the investigated systems are presented in Table 2.

Lysozyme activity assessed after 60 min incubation with reaction substrate was maintained in the presence of [Fe(CN)_6_]^3−^ but reduced in the presence of [Fe(CN)_6_]^4−^ to 82 ± 14.8% relative activity compared to lysozyme alone. Controls containing only hexacyanoferrate(III)/(II) ions or buffer showed negligible activity in the assay, confirming the specificity of the enzyme–substrate reaction.

In addition, the experimental data were fitted to the Michaelis–Menten and Lineweaver–Burk equations to obtain the kinetic parameters, specifically the maximum reaction velocity (*V*_max_) and Michaelis constant (*K*_m_) characterising the studied systems (Table 3 and Figure S4).

The *K*_m_ values determined for the investigated systems were equal to the experimental error range. Moreover, a reaction rate equal to half V_max_ was reached at a substrate concentration of around 19 µM (Table 3). The rate of 4-MUF formation by hydrolysis of 4-MUF-triNAG was 5–6 nM min^−1^ for 20 µM enzyme, irrespective of the presence of [Fe(CN)_6_]^3−/4−^ ions in the system. These results indicate that the affinity of the enzyme for the substrate remained constant, irrespective of whether free lysozyme or its complex (lysozyme-[Fe(CN)_6_]^3−/4−^) was present in the test sample.

#### 2.4.2. Biological Studies

The results of biochemical studies on lysozyme activity were subsequently verified by studying the effects of a free enzyme and lysozyme–[Fe(CN)_6_]^3−/4−^ complexes on selected bacterial strains with different morphologies. The biological tests were performed on both Gram-positive (*B. subtilis*, *S. aureus*) and Gram-negative (*E. coli*, *P. aeruginosa*) bacteria. Cacodylate buffer (used for studying binding interactions) and hexacyanoferrate(III)/(II) ions had negligible effects on bacterial strain viability. In turn, the activity of lysozyme and the lysozyme-[Fe(CN)_6_]^3−/4−^ complexes depends mainly on the type of bacteria and the experimental conditions, namely the concentration of the lytic agents and the incubation time (Figure S5). Among Gram-positive bacteria, *S. aureus* was more resistant to lysis than *B. subtilis*, which is related to various adaptations exhibited by pathogenic *S. aureus*, including modifications to peptidoglycan structure (e.g., O-acetylation or presence of teichoic acid), secretion of lysozyme inhibitors, and the presence of surface-associated proteins that enhance resistance to enzymatic degradation which may contribute to its reduced susceptibility to lysozyme [38,39]. As a soil bacterium, *B. subtilis* is less exposed to lysozyme in its natural environment and may lack such robust defence mechanisms. On the other hand, Gram-negative *E. coli* and *P. aureus* were characterised by a significant resistance to lytic agents, primarily due to their outer membrane composed of lipopolysaccharides (LPS), phospholipids, and proteins, which is impermeable to lysozyme making them inherently more resistant to lysozyme compared to Gram-positive bacteria [38,39]. Interestingly, the bioassay results show that binding hexacyanoferrate(III)/(II) ions to the protein does not change the enzyme’s activity. This finding aligns with the results from the chemical assays, indicating that [Fe(CN)_6_]^3−/4−^ ions do not inhibit lysozyme by blocking access to its active site.

## 3. Materials and Methods

### 3.1. Materials

Lysozyme from chicken egg white (dialyzed, lyophilized, powder, CAS: 12650-88-3, Sigma-Aldrich, Poland), potassium hexacyanoferrate(III) (K_3_[Fe(CN)_6_], ≥99.0%, CAS: 13746-66-2, Sigma-Aldrich, Poznań, Poland), potassium hexacyanoferrate(II) trihydrate (K_4_[Fe(CN)_6_], ≥98.5%, CAS:14459-95-1, Sigma-Aldrich, Poland), sodium cacodylate trihydrate (Caco, ≥98%, CAS: 6131-99-3, Sigma-Aldrich, Poland), sodium acetate (CH_3_COONa, ≥99.0%, CAS: 127-09-3, Sigma-Aldrich, Poznań, Poland), sodium carbonate (Na_2_CO_3_, ≥99.5%, CAS: 497-19-8, Sigma-Aldrich, Poznań, Poland), dimethyl sulfoxide (DMSO, ≥99.5%, CAS: 67-68-5, POCH, Poland), 4-methylumbelliferyl *β*-D-*N*,*N*′,*N*″-triacetylchitotrioside (4-MUF-triNAG, ≥98.0%, CAS: 53643-13-3, Sigma-Aldrich, Poznań, Poland), 4-methylumbelliferone (4-MUF, ≥98.0%, CAS: 90-33-5, Sigma-Aldrich, Poznań, Poland), potassium chloride (KCl, p.a., CAS: 7447-40-7, Stanlab, Lublin, Poland) were used without additional purification.

### 3.2. Isothermal Titration Calorimetry (ITC)

ITC experiments were performed using an Auto-ITC isothermal titration calorimeter (MicroCal, GE Healthcare, Northampton, MA, USA). Details of the instrument and experimental setup have been described previously [13]. The experimental conditions were as follows: a 10 mM cacodylate buffer; pH 7; T = 298.15 K; the sample cell containing a 0.1 mM lysozyme solution; titrations involved 2.5 mM solutions of K_3_[Fe(CN)_6_] or K_4_[Fe(CN)_6_] as the titrant; injection duration = 20 s; injection spacing = 240 s.

### 3.3. Steady-State Fluorescence Spectroscopy (SF)

All spectrofluorometric experiments were performed at 298.15 K using the stock solutions of potassium hexacyanoferrates(III)/(II) and lysozyme, which were prepared in 10 mM Caco buffer of pH 7. A Perkin Elmer Lambda 650 (Waltham, MA, USA) UV/Vis spectrophotometer was used to record the UV absorption spectra in order to spectrophotometrically measure the lysozyme concentration using an extinction coefficient equal to $\varepsilon_{280}^{LYS}$ = 38,460 $M^{-1}\;{cm}^{-1}$ [40]. Emission spectra and fluorescence intensities were recorded using the Cary Eclipse Varian (Agilent, Santa Clara, CA, USA) spectrofluorometer, which has a temperature controller and a 1.0 cm multicell holder. Both devices were calibrated with certified reference materials for wavelength and absorbance to ensure the accuracy of the measurements. The widths of the excitation and emission slits were set at 5 nm. The constant excitation wavelength was 275 nm. The inner filter effect was adjusted for all fluorescence intensity values. In the absence and presence of increasing concentrations of K_3_[Fe(CN)_6_] and K_4_[Fe(CN)_6_], each up to 9.76 µM, the fluorescence emission spectra of lysozyme (1.76 µM) were recorded from 290 to 500 nm. Ten additions of 2 µL and then six additions of 5 µL stock solutions of K_3_[Fe(CN)_6_] and K_4_[Fe(CN)_6_] (c = 0.4 mM) were used to titrate 2 mL of the protein at a fixed concentration equal to 1.76 µM in the subsequent fluorescence titration experiments (conducted manually using a micropipette). The mixture was shaken after each addition, and at least ten readings of the fluorescence intensity were taken. The binding constants and other parameters were determined using the fluorescence intensities of the band at 345 nm, which corresponds to the initial maximum emission of lysozyme.

### 3.4. Circular Dichroism Spectroscopy (CD)

Circular dichroism (CD) spectra were recorded at 298.15 K using a Jasco-715 automated spectropolarimeter (Jasco Inc., USA). The analysed solutions contained 14 μM lysozyme and a ligand (either [Fe(CN)_6_]^3−^ or [Fe(CN)_6_]^4−^) at lysozyme-to-ligand molar ratios of 1:0, 1:1, 1:5 and 1:10. All solutions were prepared in the 10 mM Caco buffer of pH 7. The experimental data were processed according to the procedure described in [41].

### 3.5. Molecular Dynamics (MD) Simulations

The structure of the wild-type chicken lysozyme was obtained from the PDB (PDB ID: 193L, 1.33 Å). Details of the structures and calculations involving [Fe(CN)_6_]^3−^ and [Fe(CN)_6_]^4−^ ions are described in [13]. Briefly, MD simulations were conducted using AMBER20 [42] for two systems: lysozyme with 10 hexacyanoferrate(III) and hexacyanoferrate(II) species. Ligands were randomly placed around the protein in a truncated octahedron TIP3P water box with a 10 Å water layer, and 22 and 32 K^+^ ions were added to the respective systems. The ff14SB force field [43] was used for the protein, and gaff [44] with custom parameters was used for the cyanide complexes. The systems underwent two energy minimization steps—first with restraints, then without—followed by heating from 0 to 300.15 K over 10 ps with restraints. Equilibration was performed at 300.15 K and 105 Pa for 100 ps, followed by a 0.5 μs production run performed in the same isothermal isobaric ensemble. The particle mesh Ewald method for treating electrostatics and the SHAKE algorithm for all the covalent bonds containing hydrogen atoms were applied. The analysis of the obtained trajectories was performed with the ccptraj module of AMBER 20, the visualisation and statistical analysis were conducted in VMD [45] and R package [46], respectively.

### 3.6. Biochemical and Biological Assays

*Lysozyme Activity Assay*. The enzymatic activity of lysozyme was quantified using a fluorometric assay with a synthetic substrate for the enzyme. The protocol was developed based on previous studies [47,48]. The reagents and solutions included 20 mM sodium acetate at pH 5 (assay buffer), 0.2 M sodium carbonate at pH 10–11 (stop buffer), 10 mM 4-methylumbelliferyl *β*-D-*N*,*N*′,*N*″-triacetylchitotrioside (4-MUF-triNAG) hydrate prepared in dimethyl sulfoxide (substrate stock solution; stored in aliquots at 253.15 K protected from light), and 5 mM 4-methylumbelliferone (4-MUF) prepared in dimethyl sulfoxide (standard stock solution; stored in aliquots at 253.15 K protected from light).

Reactions were performed in 96-well black plates at a final volume of 100 µL per well. Test samples (20 µL) were mixed with 70 µL of assay buffer and 10 µL of substrate working solution (1 mM 4-MUF-triNAG prepared freshly by diluting the substrate stock solution in assay buffer immediately before use). Sample background controls (20 µL sample and 80 µL assay buffer) and reagent background controls (90 µL assay buffer and 10 µL substrate working solution) were included. Standards were prepared by serial dilution of 10 µM 4-MUF working solution (freshly prepared in assay buffer from 4-MUF stock solution) to generate a standard curve (0–200 pmol/well). The reactions were incubated at 310.15 K for 60 min in the dark and terminated by adding 100 µL of stop buffer. Fluorescence was measured immediately at excitation/emission wavelengths of 360/445 nm using a microplate reader.

The corrected fluorescence was determined by subtracting the blank readings from all the samples and standard readings. A standard curve was plotted, and the amount of 4-MUF generated in the test samples was calculated using the standard curve equation. The lysozyme activity (pmol min^−1^ mL^−1^) was determined using the following formula:

(4) $$lysozyme\;activity=\left(\frac{B}{∆T\times V}\right)\times D,$$

where *B* is the amount of 4-MUF (pmol) in the sample wells, Δ*T* is the reaction time (min), *V* is the sample volume (mL), and *D* is the dilution factor.

Lysozyme Kinetic Assay. The enzyme kinetic was assessed as described above, with reaction mixtures containing 4-MUF-triNAG substrate concentrations ranging from 1.5625 to 100 µM, prepared by twofold serial dilutions, and a fixed enzyme concentration of 20 µM. Reactions were terminated at 5 min intervals by the addition of stop buffer. The amount of 4-MUF generated was calculated using the standard curve described above, and these values were used to determine the kinetic parameters using the online tool ICEKAT [49,50].

Bacterial Lysis Assay. The effects of the tested compounds on bacterial lysis were evaluated using the broth dilution method. The bacterial strains used were *Bacillus subtilis*, *Escherichia coli*, *Staphylococcus aureus*, and *Pseudomonas aeruginosa*. Overnight cultures of these strains were grown in Luria–Bertani (LB) broth at 310.15 K with shaking. For inoculation, bacterial suspensions were prepared by diluting overnight cultures in LB broth to an optical density at 600 nm (OD600) of 0.6.

In a 96-well plate, 100 μL of LB broth containing two-fold serial dilutions of the tested compounds (final concentration range: 3.13–25 μM) was combined with 100 μL of the prepared bacterial suspensions. Background controls consisted of LB broth with the tested compounds, but without bacterial inoculation. Plates were incubated at 310.15 K with shaking, and bacterial growth was monitored by measuring the OD600 at 1 and 18 h using a microplate reader.

Bacterial lysis activity was calculated as the percentage of lysis relative to the growth of the control (bacteria in LB broth without compounds), based on OD600 values corrected for background controls.

Statistical Analysis. One-way ANOVA followed by Tukey’s post hoc test was performed to assess statistical significance, with a threshold of *p* < 0.05 considered significant.

### 3.7. Cyclic Voltammetry (CV)

The cyclic voltammetry (CV) was carried out using a Multi Autolab M204, by Metrohm. All measurements were performed in three electrode cells at room temperature, using a glassy carbon electrode as the working electrode, a platinum wire as the counter electrode and Ag/AgCl in 0.1 M NaCl as the reference electrode. The working electrode of diameter 3 mm before each experiment was polished on polishing pads (Buehler) using 3 µm alumina suspension. All cyclic voltammograms were obtained in degassed solutions by passing argon through them for 5 min, at a scan rate of 100 mV s^−1^.

## 4. Conclusions

A few complementary experimental methods supported by in silico analysis have successfully been applied to describe the physicochemical nature of lysozyme interactions with a model highly charged species, namely [Fe(CN)_6_]^3−^ and [Fe(CN)_6_]^4−^. Special attention was paid to the understanding of the physicochemical principles underlying these interactions, in particular the factors influencing the mutual affinity between the studied molecules and the nature of the intermolecular forces involved. In addition, the impact of these interactions on the biological activity of the enzyme and the redox properties of [Fe(CN)_6_]^3−/4−^ ions has been assessed. Lysozyme–[Fe(CN)_6_]^3−/4−^ complexes have been shown to exhibit moderate stability compared to other known protein–ligand complexes. In particular, their formation does not interfere with enzyme activity, as confirmed by biochemical assays and supported by molecular dynamics simulations. These findings suggest that hexacyanoferrate(III)/(II) ions do not hinder access to the active site of the enzyme, thus allowing its proper catalytic activity. This comprehensive approach is advancing our knowledge of the mechanisms governing enzyme–ligand interactions and their implications for both biochemical function and potential applications. Furthermore, the finding that [Fe(CN)_6_]^3−/4−^ ions do not interfere with the active site of lysozyme suggests their potential role as cofactors in enzymatic reactions, which justifies further investigation of their mechanistic contributions in biological systems.

## Figures and Tables

**Table 1:** The conditional thermodynamic parameters of K_3_[Fe(CN)_6_] and K_4_[Fe(CN)_6_] binding to lysozyme (standard deviation values in parentheses) in the 10 mM Caco buffer of pH 7, at 298.15 K.

Parameter	Lysozyme	
	K_3_[Fe(CN)_6_]	K_4_[Fe(CN)_6_]
*n* (*number of binding sites*)	1.03	1.10
*N* ** (*stoichiometry*)	1.01 (±0.01)	0.86 (±0.02)
log(*K*_SV_) *^1^	4.29	5.06
log(*K*) *^2^	4.27	4.84
log(*K*) *^3^	4.43	5.59
log(*K)* **	3.90 (±0.02)	4.56 (±0.03)
Δ*G* ** [kJ mol^−1^]	−22.30 (±0.13)	−26.02 (±0.17)
Δ*H* ** [kJ mol^−1^]	−16.61 (±0.29)	−19.62 (±0.59)
TΔ*S* ** [kJ mol^−1^]	5.69	6.40

*^1^ SF data (Equation (1)); *^2^ SF data (Equation (2)); *^3^ SF data (Equation (3)); ** ITC data.

**Table 2:** Enzymatic activity of the lysozyme systems investigated. The activity was assessed after 60 min of incubation with a synthetic substrate at 310.15 K. Each reaction contained 2 nmoles of enzyme and 10 nmoles of substrate (equivalent to 100 µM) in a final volume of 100 µL. *N*, number of replicates.

Reagent	*N*	Activity (Moles min^−1^ mL^−1^)	Activity (% Vs. Lysozyme)
lysozyme	5	21.9 (±1.20) ^a^	100 (±5.5)
lysozyme-[Fe(CN)_6_]^3−^	4	22.0 (±1.59) ^b^	101 (±7.3)
lysozyme-[Fe(CN)_6_]^4−^	4	17.8 (±3.24) ^ab^	82 (±14.8)
[Fe(CN)_6_]^3−^	3	0.3 (±0.13)	1 (±0.6)
[Fe(CN)_6_]^4−^	3	0.2 (±0.19)	1 (±0.9)
Caco buffer	3	0.0 (±0.00)	0 (±0.0)

^a,b^—significant difference between means with common superscript determined by ANOVA (F(2,10) = 5.3067, *p* = 0.0269) and Tukey’s post hoc test (*p* < 0.05) performed for lysozyme-containing systems.

**Table 3:** Enzyme kinetic coefficients for the investigated systems. Values were calculated from data obtained from two independent experiments. [E], enzyme concentration; *K*_m_, Michaelis constant; *V*_max_, maximal velocity (maximal reaction rate); *k*_cat_, catalytic constant; *k*_cat_/*K*_m_, specificity constant (catalytic efficiency).

Coefficient	Unit	Lysozyme	Lysozyme- [Fe(CN)_6_]^3−^	Lysozyme-[Fe(CN)_6_]^4−^
[E]	µM	20	20	20
*K*_m_	µM	19.12 (±0.40)	18.57 (±3.29)	19.04 (±2.15)
*V*_max_	nM min^−1^	5.39 (±0.23)	5.89 (±0.37)	5.28 (±0.21)
*k*_cat_	min^−1^	0.270	0.295	0.264
*k*_cat_/*K*_m_	nM^−1^ min^−1^	0.014	0.016	0.014

No significant differences in *K*_m_ and *V*_max_ were detected by ANOVA, with *F*(2,3) = 0.0341 (*p* = 0.9668) and *F*(2,3) = 2.7002 (*p* = 0.2134), respectively.

## Data Availability

Data are contained within the article and Supplementary Materials.

## Supplementary Materials

The following supporting information can be downloaded at: https://www.mdpi.com/article/10.3390/ijms26178511/s1.
