# Supplementary material for: An Interdisciplinary Study of Lysozyme Interactions with Hexacyanoferrate(III)/(II) Ions
Source: Int J Mol Sci. 2025 Sep 2;26(17):8511. doi: 10.3390/ijms26178511 (PMC12428812; doi:10.3390/ijms26178511)
Supplement: Supplementary file 1 [file ijms-26-08511-s001.zip › ijms-3844707-supplementary.pdf]

Electronic Supplementary Information

An interdisciplinary study of lysozyme interactions with hexa-cyanoferrate(III)/(II) ions

Ola Grabowska[1]*, Krzysztof Żamojć[1], Anna Kloska[2], Paweł Niedziałkowski[1], Sergey A. Samsonov[1], Dariusz Wyrzykowski[1]*

[1]Faculty of Chemistry, University of Gdańsk, Wita Stwosza 63, 80-308 Gdańsk, Poland

[2]Faculty of Biology, University of Gdańsk, Wita Stwosza 59, 80-308 Gdańsk, Poland

*Corresponding authors:

E-mail address: **ola.grabowska@ug.edu.pl; dariusz.wyrzykowski@ug.edu.pl**

telephone number: (+48 58) 523 50 57,

Contents

Figure S1. CD spectra of lysozyme and the mixture of lysozyme-$[Fe(CN)_6]^{3-/4-}$ in a molar ratio of 1:10, in the 10 mM Caco buffer at pH 7 and 298.15 K.

Figure S2. Cyclic voltammogram of 1 mM $[Fe(CN)_6]^{3-/4-}$ in 0.1 M KCl (**A**); Cyclic voltammogram of 0.93 mM $[Fe(CN)_6]^{3-/4-}$ in the 0.1 M Caco buffer, pH 7 (**B**); Cyclic voltammogram of 0.1 M Caco buffer, pH 7 (**C**); Cyclic voltammogram of 0.93 mM $[Fe(CN)_6]^{3-/4-}$ with the addition of 3.73 mM lysozyme in the 0.1 M Caco buffer, pH 7 (**D**). All experiments were obtained at glassy carbon electrode, scan rate of 100 mV s^{-1}.

Figure S3. Number of contacts per frame for hexacyanoferrate(III) ion ($[Fe(CN)_6]^{3-}$, x-axis) and hexacyanoferrate(II) ion ($[Fe(CN)_6]^{4-}$, y-axis). Contacts are defined by the cut-off of 10 Å between the center of mass of a ligand molecule and any protein residue.

Figure S4. Michaelis-Menten (left) and Lineweaver-Burk (right) plots for the investigated systems. Values represent the mean from two independent experiments. Lys, lysozyme; Lys-K3, lysozyme-$[Fe(CN)_6]^{3-}$; Lys-K4, lysozyme-$[Fe(CN)_6]^{4-}$; [S], substrate concentration; V_0, initial reaction rate.

Figure S5. Lysis of bacterial cultures treated with lytic agents. The percentage of lysed cells was calculated relative to the growth control (bacteria grown in LB broth without lytic agents). Lysis of Gram-positive (*Bacillus subtilis*, *Staphylococcus aureus*) and Gram-negative

(*Escherichia coli*, *Pseudomonas aeruginosa*) bacteria was assessed after 1 h and 18 h of incubation with lytic agents. Data are presented as mean ± standard deviation from three independent experiments.

Table S1. The secondary structure content [%] of lysozyme in the absence and presence of $[Fe(CN)_6]^{3-}$ and $[Fe(CN)_6]^{4-}$ complex, in the 10 mM Caco buffer at pH 7 and 298.15 K.

FIGURES

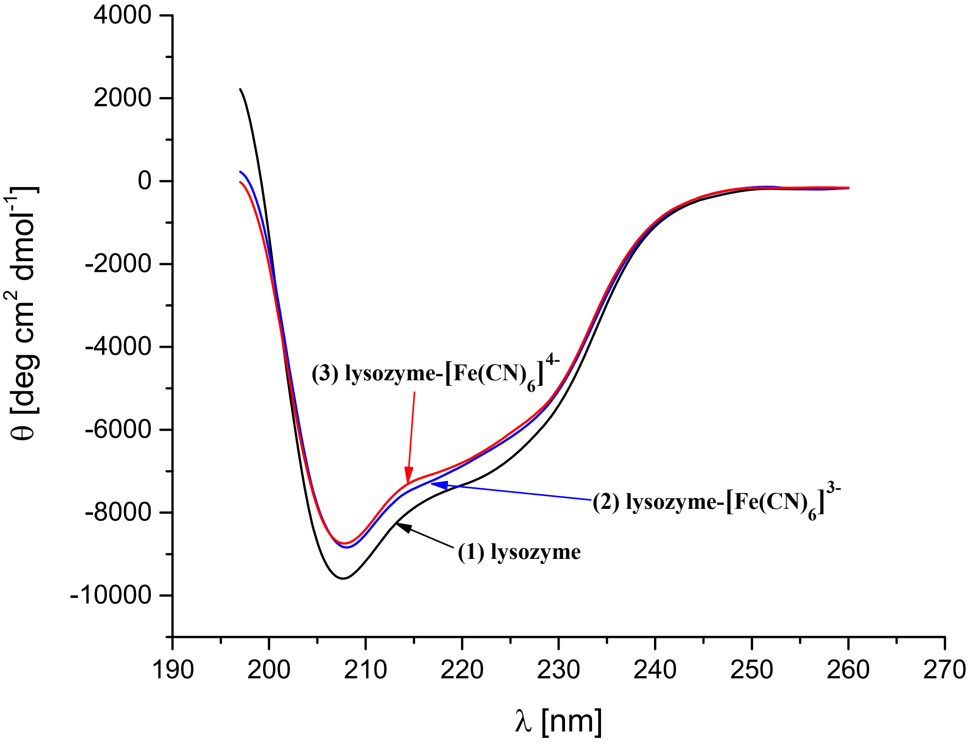

Figure S1. CD spectra of lysozyme and the mixture of lysozyme-$[Fe(CN)_6]^{3-/4-}$ in a molar ratio of 1:10, in the 10 mM Caco buffer at pH 7 and 298.15 K.

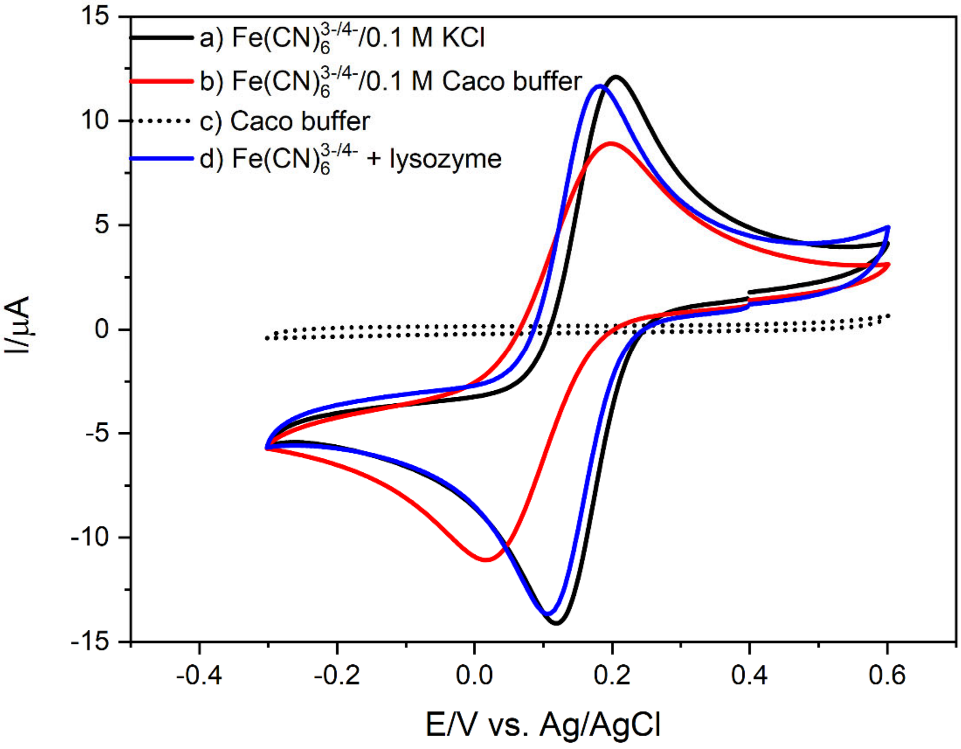

Figure S2. Cyclic voltammogram of 1 mM $[Fe(CN)_6]^{3-/4-}$ in 0.1 M KCl (**A**); Cyclic voltammogram of 0.93 mM $[Fe(CN)_6]^{3-/4-}$ in the 0.1 M Caco buffer, pH 7 (**B**); Cyclic voltammogram of 0.1 M Caco buffer, pH 7 (**C**); Cyclic voltammogram of 0.93 mM $[Fe(CN)_6]^{3-/4-}$ with the addition of 3.73 mM lysozyme in the 0.1 M Caco buffer, pH 7 (**D**). All experiments were obtained at glassy carbon electrode, scan rate of 100 mV s^{-1}.

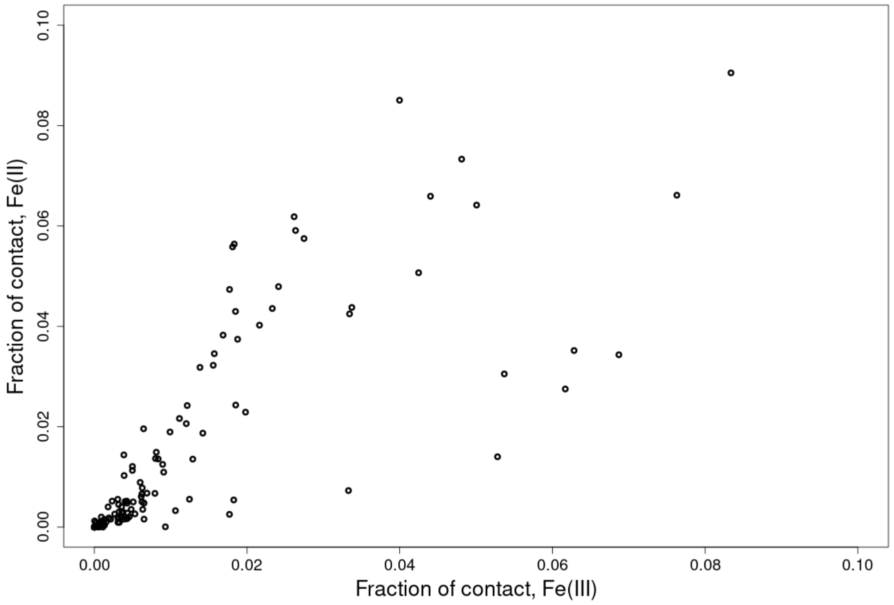

Figure S3. Number of contacts per frame for hexacyanoferrate(III) ion ($[Fe(CN)_6]^{3-}$, x-axis) and hexacyanoferrate(II) ion ($[Fe(CN)_6]^{4-}$, y-axis). Contacts are defined by the cut-off of 10 Å between the center of mass of a ligand molecule and any protein residue.

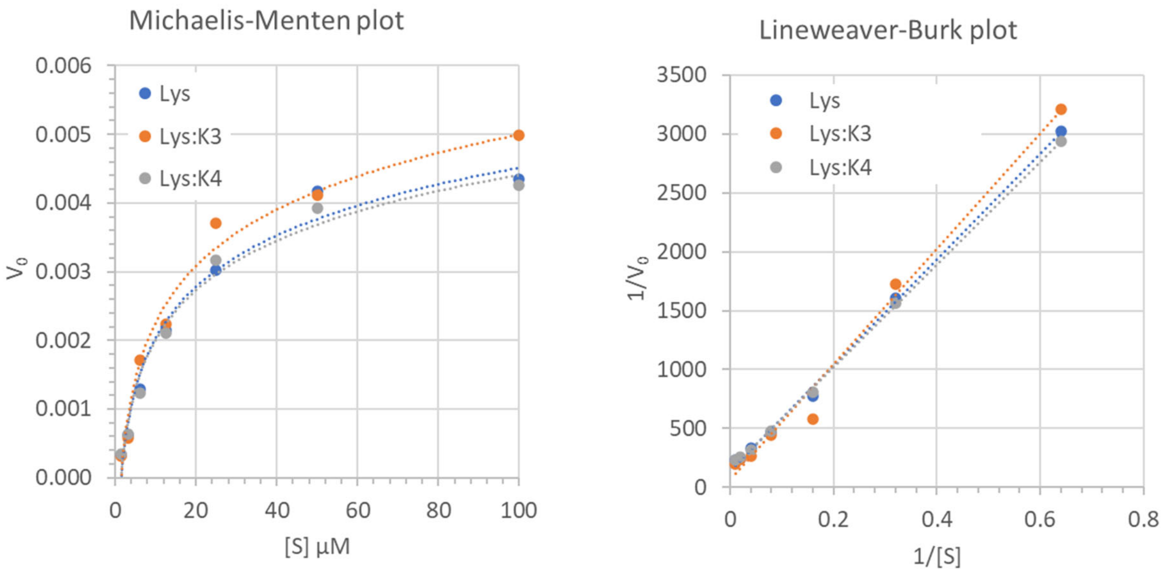

Figure S4. Michaelis-Menten (left) and Lineweaver-Burk (right) plots for the investigated systems. Values represent the mean from two independent experiments. Lys, lysozyme; Lys-K3, lysozyme-$[Fe(CN)_6]^{3-}$; Lys-K4, lysozyme-$[Fe(CN)_6]^{4-}$; [S], substrate concentration; V_0, initial reaction rate.

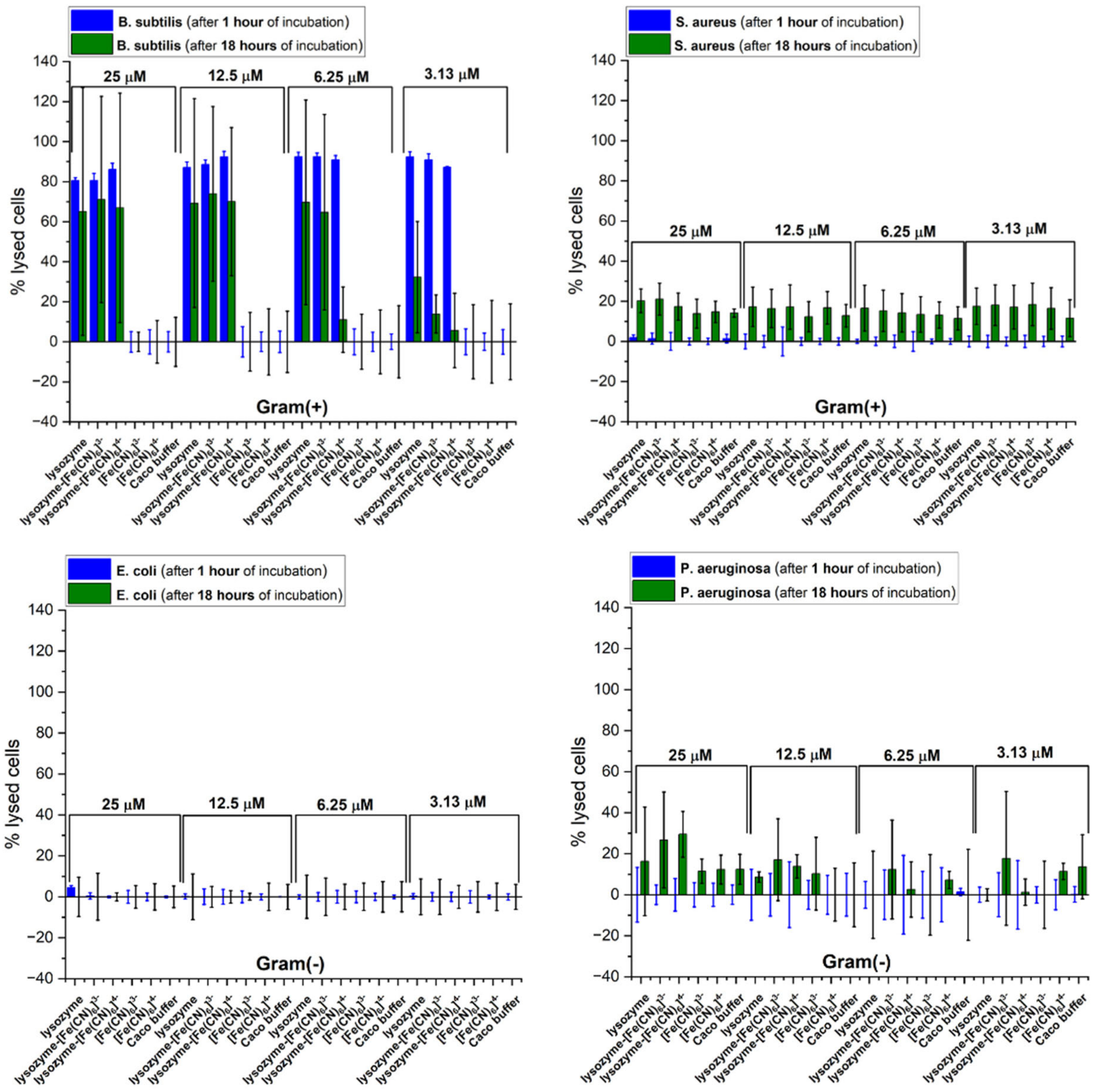

Figure S5. Lysis of bacterial cultures treated with lytic agents. The percentage of lysed cells was calculated relative to the growth control (bacteria grown in LB broth without lytic agents). Lysis of Gram-positive (*Bacillus subtilis*, *Staphylococcus aureus*) and Gram-negative (*Escherichia coli*, *Pseudomonas aeruginosa*) bacteria was assessed after 1 h and 18 h of incubation with lytic agents. Data are presented as mean ± standard deviation from three independent experiments.

TABLE

Table S1. The secondary structure content [%] of lysozyme in the absence and presence of $[Fe(CN)_6]^{3-}$ and $[Fe(CN)_6]^{4-}$ complex, in the 10 mM Caco buffer at pH 7 and 298.15 K.

**System: lysozyme-$[Fe(CN)_6]^{3-}$**				
molar ratio (protein:ligand)	The percentage content of [%]			
	α-helix	*β*-structure	*β*-turn	random coil
1:0	23	18	17	42
1:1	23	18	18	42
1:5	23	18	16	44
1:10	20	18	17	45
**System: lysozyme-$[Fe(CN)_6]^{4-}$**				
molar ratio (protein:ligand)	The percentage content of [%]			
	α-helix	*β*-structure	*β*-turn	random coil
1:0	23	18	17	42
1:1	21	18	17	43
1:5	21	18	16	46
1:10	20	18	16	46
